# Association of Cerebral Artery Stenosis With Post-stroke Depression at Discharge and 3 Months After Ischemic Stroke Onset

**DOI:** 10.3389/fpsyt.2020.585201

**Published:** 2020-11-25

**Authors:** Xiuli Qiu, Jinfeng Miao, Yan Lan, Wenzhe Sun, Yuxi Chen, Ziqin Cao, Guo Li, Xin Zhao, Zhou Zhu, Suiqiang Zhu

**Affiliations:** ^1^Department of Neurology, Tongji Medical College, Tongji Hospital, Huazhong University of Science and Technology, Wuhan, China; ^2^The Solomon H. Snyder Department of Neuroscience, Johns Hopkins University School of Medicine, Baltimore, MD, United States; ^3^Emory University, Emory University, Atlanta, GA, United States

**Keywords:** post-stroke depression, cerebral artery diseases, stroke, nomogram, prediction

## Abstract

**Background:** Post-stroke depression (PSD) is one of the most common complications after stroke, which seriously affects patients' recovery outcome. Although vascular depression has been extensively studied, the relationship between cerebral artery stenosis and PSD has not been clarified so far.

**Methods:** Two hundred ninety-eight patients with ischemic stroke (72 women, 226 men) with computed tomography angiography (CTA) or magnetic resonance angiography (MRA) were included in this study. Cerebral artery stenosis ≥50% was used as the cut-off value. The DSM-V diagnostic criteria of PSD was met and the 17-item Hamilton Rating Scale for Depression (HAMD-17) score over 7 at discharge and 3 months after stroke onset was regarded as the primary outcome. The χ^2^-test, Mann-Whitney *U*-test, and *t*-test were used to check for statistical significance.

**Results:** At discharge, Barthel index (*p* < 0.001), left middle cerebral artery stenosis (*p* = 0.019), drinking history (*p* = 0.048), basilar artery stenosis (*p* = 0.037) were significantly associated with PSD. At 3 months after ischemic stroke onset, Barthel index (*p* = 0.011), left middle cerebral artery stenosis (*p* = 0.012), female gender (*p* = 0.001) were significantly associated with PSD.

**Conclusions:** The findings demonstrated that left middle cerebral artery and basilar artery stenosis are associated with PSD. It was suggested that cerebral artery stenosis was a risk factor of PSD and should be recognized and intervened early.

**Registration Number:** ChiCTR-ROC-17013993.

## Introduction

Post-stroke depression (PSD), characterized by obvious reduction in mood and physical vitality (1–3), is a common psychiatric comorbidity after stroke. In the first few months after stroke, the incidence of PSD fluctuates around 33–40% among stroke survivors (4, 5). PSD negatively impacts motor skill recuperation, aggravates cognitive deficits, and increases the risk of stroke recurrence and death. At the same time, it also exerts a negative effect on the quality of life of survivors and brings heavy burden to caregivers. Therefore, early detection of PSD high-risk groups as well as targeted preventive and treatment measures are particularly important to improve the prognosis of PSD patients.

So far, the pathophysiological mechanism of PSD is unclear (6). Evidence from neuropsychology, genetics, and epidemiology suggests that cerebrovascular changes may cause PSD (7, 8). PSD was also found to be associated with stroke lesions in the left frontal lobe (9, 10). Moreover, previous studies have shown that severe internal carotid artery stenosis can cause depression (11), and treatment with internal carotid artery stenting can improve depressive symptoms (12). Studies have also shown that the hemodynamics changes of the middle cerebral artery are related to the onset of depression in the elderly (13). In addition, animal studies have shown that depression-like behavior in rats was related to cerebral hypoperfusion (14). It has been well-established that artery stenosis can cause hemodynamic disorders and hypoperfusion. The patient with cerebral artery stenosis may have experienced corresponding cerebral hypoperfusion for a long time before the onset of ischemic stroke. However, it has not been studied whether depression after ischemic stroke was related to cerebral artery stenosis.

The main purpose of this study was to evaluate whether cerebral artery stenosis in patients with ischemic stroke measured by computed tomography angiography (CTA) or magnetic resonance angiography (MRA) was related to PSD at discharge and 3 months after the attack. Specifically, nomogram based on multiple logistic regression analysis or Cox regression model was widely used as a convenient clinical tool for risk assessment of clinical events of interest (15, 16). Therefore, the study intends to construct a convenient nomogram screening tool that integrates the predictors of clinical characteristics and location of cerebral artery stenosis to predict PSD at discharge and 3-month after ischemic stroke onset.

## Methods

This was a prospective cohort study (Registration number: ChiCTR-ROC-17013993) in the department of neurology, Tongji Hospital, Huazhong University of Science and Technology. The study recruited 298 patients with consecutive acute ischemic stroke (AIS) admitted to the department of neurology of Tongji hospital from August 2018 to May 2019. The inclusion criteria were: (1) ages 18 years and over; (2) admitted to hospital within 7 days after ischemic stroke onset; (3) AIS patients confirmed by computed tomography (CT) or magnetic resonance imaging (MRI) scans. Exclusion criteria were: (1) brain tumors, metastatic encephaloma, brain trauma, and other non-vascular causes resulted in brain disorder; (2) the history of depression, antidepressants, dementia, and other mental illness before ischemic stroke; (3) aphasia, deafness, blindness, and cognitive dysfunction (a Mini-Mental State Examination score <17 points in people with less than primary school education); (4) failure to complete follow-up; (5) transient ischemic attack (TIA) and subarachnoid hemorrhage. This study was approved by the ethics committee of Tongji Medical College, Huazhong University of Science and Technology (Approved No. of ethic committee: TJ-IRB20171108). According to the Declaration of Helsinki, all subjects signed an informed consent form.

### Data Collection

Upon admission, a standardized questionnaire was used to collect detailed information about each patient's demographic and medical history, including age, gender, education level, smoking history, drinking history, hypertension, diabetes mellitus, hyperlipidemia, coronary artery disease, history of stroke, and treatment of stenosis (thrombolysis/thrombectomy/stents implantation). In our study, drinking history and smoking history were binary variables, no-smoker, and no drinking were regard as no smoking and drinking history, others were considered to have smoking and drinking history (17, 18). The National Institutes of Health Stroke Scale (NIHSS), Barthel index (BI), Modified Rankin scale (MRS), Social Support Rating Scale (SSRS), and the 17-item Hamilton Rating Scale for Depression (HAMD-17) were evaluated by two qualified and formally trained doctors (X.S. and W.S) at discharge and 3 months after ischemic stroke onset. MRA or CTA was completed during hospitalization.

PSD was diagnosed by a psychiatrist who was blinded to the study at discharge and 3 months after the onset of ischemic stroke. HAMD-17 was used to measure the severity of depressive symptoms. The DSM-V PSD diagnostic criteria (depression caused by other medical conditions) was met and the HAMD-17 score over 7 (19–22) at discharge and 3 months after ischemic stroke onset was used as the primary outcome.

### MRA and CTA Protocol

A total of 298 patients completed CTA or MRA, of which 84 patients were scanned on a GE 3.0T MR scanner (Discovery MR 750 System, GE Healthcare, Milwaukee, WI, USA) with an 8-channel head coil. 3D Time-of-flight MRA was obtained using TR/TE = 19/3.4 ms, FOV = 31.4 × 22 cm, thickness = 1.2 mm, matrix = 256 × 256, and NEX = 1, number of slices = 160. Two hundred fourteen patients underwent CTA scanning from the aortic arch to vertex with the Discovery CT750 HD scanner (HDCT, GE Healthcare, Milwaukee, WI, USA). In brief, non-enhanced CT brain scan was first performed, followed by contrast enhanced CTA. The CTA images were obtained when contrast agent (Iopromide 370, Bayer Schering Pharma AG) with a dose of 1.0 ml/kg was injected at a rate of 5 ml/s, followed by 40 ml of saline chase injected intravenously at a rate of 5 ml/s. Standard 3-dimensional CT angiography scanning parameters were used. The slice thickness was 0.63 mm, section interval was 0.3 mm, the helical pitch was 1, 26.8 cm × 25 cm DFOV (display field of view), 80 kVp and 400 mAs, rotation time of 0.5 s.

### Imaging Review

The degree of stenosis was estimated on source image or maximum intensity projection (MIP) images of CTA by the diameter stenosis. The measurement method refers to the North American Symptomatic Carotid Endarterectomy Trial (NASCET) (23). Two professional neurologists (X.Q. and J.M) independently and quantitatively measured diameters on coronal, sagittal, or horizontal. In order to maintain consistency in the selection of vessel wall locations, the narrowest vessel was selected on both CT angiographic and source images. The cerebral arterial system evaluated included bilateral middle cerebral artery M1 segment, bilateral internal carotid artery C1 segment and basilar artery. An internationally standardized equation was used to calculate the degree of stenosis. Percent stenosis = [1 – (D stenosis/D normal)^*^100] (D Stenosis: the diameter of the most severe stenosis; D normal: the diameter of the proximal normal artery). Artery stenosis was defined as the diameter of arterial lumen below 50%. In patients who underwent MR angiography (MRA), the degree of stenosis was measured by MRA. Measurements were also taken by the two professional neurologists (X.Q. and J.M). The measurement, calculation and classification method were the same for CTA. Cerebral atrophy was evaluated by Ventricle-to-brain ratio (VBR) (24), VBR = [(width of anterior horns of lateral ventricle/corresponding brain width at the same level) + (biventricular width at the level of the body of caudate nucleus/corresponding brain width at the same level) + (width of occipital horns of lateral ventricle/corresponding brain width at the same level)]/3. CT, MRI, or DWI (Diffusion Weighted Image) confirmed the location of lesion and counted all lesions when the patient had multi-site cerebral infarctions.

### Statistical Analysis

Data analysis were performed using the Statistical Program for Social Sciences (SPSS) statistical software (version 25, Chicago, IL, USA). Intraclass correlation coefficient (ICC) was used to determine the interobserver consistency for the measurements of the degree of artery stenosis and the HAMD-17 score. Continuous data were described by the median and IQR (25th−75th percentile) or mean with standard deviation and compared using *t*-test or Mann–Whitney *U*-test (when continuous variables had skewed distributions). The dichotomous data were described by the number and percentage of pages and compared using χ^2^-test. Every variable was analyzed by univariate analysis to cover all potentially important predictors. Variables with *P* ≤ 0.10 in univariate analysis were included in multivariable logistic regression analysis and with *p* < 0.05 considered statistically significant.

Based on the relevant factors, a nomogram to predict PSD was established at discharge and 3 months after ischemic stroke onset. The nomogram was based on the results of multivariate logistic regression analysis and the R package “rms” in R software version 3.5.1 (http://www.r-project.org/). The nomogram was based on the ratio of converting each regression coefficient in multiple logistic regression to 0–100-point scale. The effect of the variable with the highest β coefficient (absolute value) was assigned 100 points. The predictive performance of the nomogram was measured by the concordance index (C index) equivalent to area under the curve value (AUC), and calibrated with 200 bootstrap samples. A higher C-index indicates better ability to separate PSD patients with different depression risk. The calibration curves are used to compare the predicted and observed probabilities in the study. If the model was calibrated correctly, the dots on the calibration plot should be close to a 45° diagonal line.

## Results

**Figure 1:**
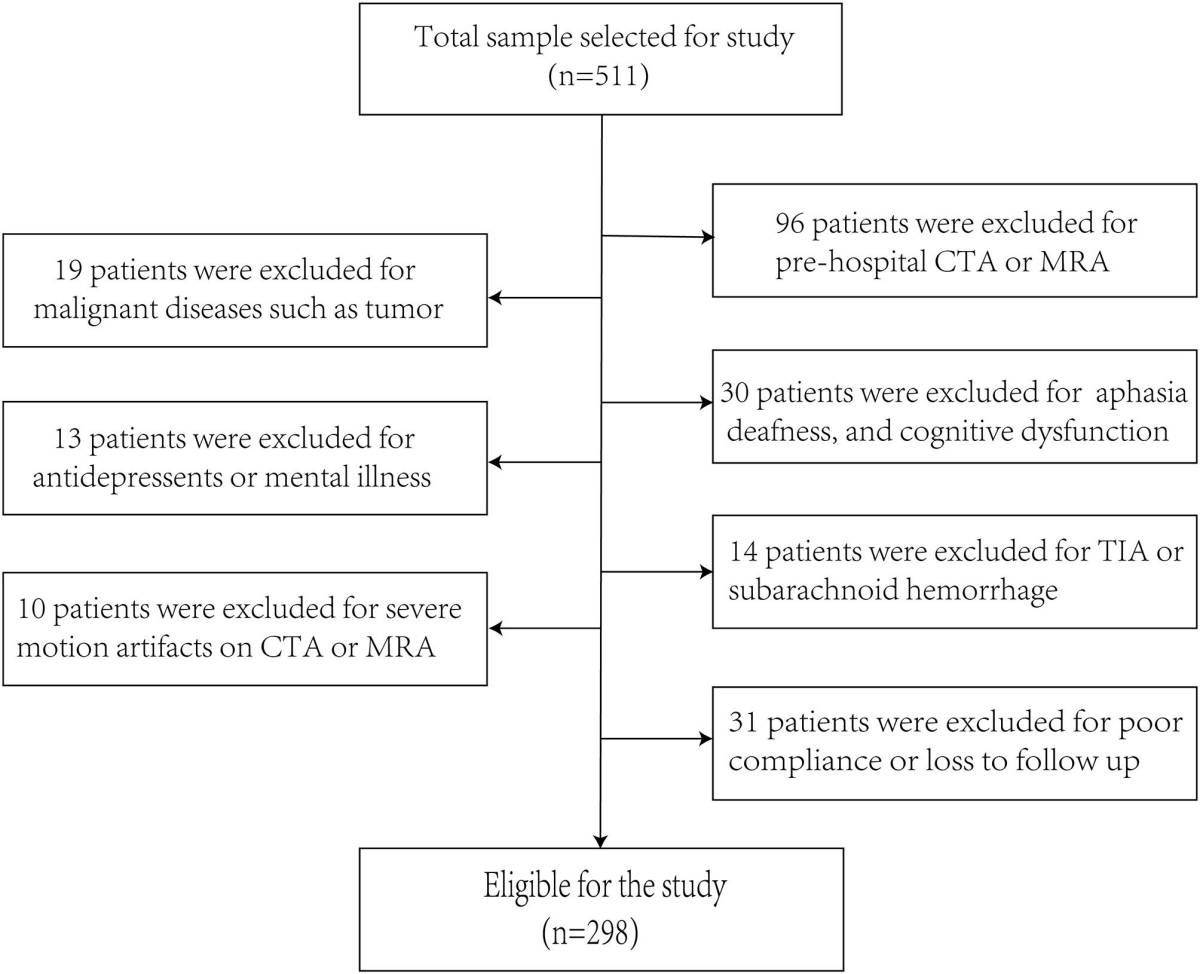
The enrollment flow chart of this study. A flowchart drawn according to inclusion and exclusion criteria.

A total of 511 patients participated in this study, and 298 patients who met the inclusion criteria formed the study sample (Figure 1). The age of the enrolled patients was 57.42 ± 10.15 (mean ± SD), 75.84% of patients were male and 24.16% of patients were female, 40.27% patients had smoking history, 16.44% patients had drinking history, 54.36% patients had a history of hypertension, and 24.83% patients had a history of diabetes mellitus. The proportion of patients with low, medium, and high education level were 33.22, 53.69, and 13.09%. All patients were admitted 4.24 ± 2.23 days after onset of ischemic stroke, and the time of hospitalization was 12.53 ± 6.43 days. In addition, two patients were on antiepileptics for post-stroke epilepsy, 28 were on beta blockers for hypertension and the proportion of PSD patients receiving antidepressant treatment at discharge and 3 months was 28.2% (37/131 = 0.282) and 30.0% (40/133 = 0.300), respectively. In our study, the percentage of patients receiving neurorehabilitative treatment after discharge was 23.5% (70/298 = 0.235).

Table 1 shows a comparison of baseline information between the PSD and non-PSD groups at discharge and 3 months after ischemic stroke onset. At discharge, the PSD group had a lower proportion of drinking history (*p* = 0.036), higher NIHSS score (*p* < 0.001), lower BI score (*p* < 0.001), higher MRS score (*p* < 0.001), higher ratio of left middle cerebral artery stenosis (*p* = 0.008), and basilar artery stenosis (*p* = 0.019). At 3 months after the onset of ischemic stroke, compared with non-PSD patients, the PSD patients had higher proportion of female (*p* < 0.001), higher NIHSS score (*p* = 0.001), lower BI score (*p* < 0.001), higher MRS score (*p* < 0.001), and higher proportion of left middle cerebral artery stenosis (*p* = 0.011). Table 2 shows the relationship between lesion location and PSD in 298 patients, among which 83 patients had multi-site cerebral infarction, and each lesion location was counted for these patients. No statistical significance was found (*p* > 0.05). The measurements of CTA (ICC = 0.905; 95% CI: 0.661–0.976), MRA (ICC = 0.885; 95% CI: 0.602–0.970) and HAMD-17 score (ICC = 0.917; 95% CI: 0.790–0.967) had high interobserver consistency.

**Table 1:** Comparisons of demographic and clinical variables.

**Variables**	**At discharge**			**At 3 months**		
	**PSD (131)**	**Non-PSD (167)**	***P*-value**	**PSD (133)**	**Non-PSD (165)**	***P*-value**
Age (years), mean ± SD	58.29 (10.22)	56.77 (10.06)	0.208	57.62 (9.94)	57.25 (10.33)	0.977
Female, *n* (%)	34 (26)	38 (22.8)	0.522	47 (35.3)	25 (15.2)	0.000
Education level, *n* (%)			0.057			0.536
Low	53 (40.5)	46 (27.5)		48 (36.1)	51 (31)	
Medium	64 (48.9)	96 (57.5)		70 (52.6)	90 (54.5)	
High	14 (10.7)	25 (15)		15 (11.3)	24 (14.5)	
Smoking history, *n* (%)	48 (36.6)	73 (44)	0.201	51 (38.3)	70 (42.7)	0.449
Drinking history, *n* (%)	15 (11.5)	34 (20.5)	0.036	20 (15.0)	29 (17.7)	0.541
Hypertension, *n* (%)	75 (57.3)	87 (52.1)	0.375	76 (57.1)	86 (52.1)	0.387
Diabetes mellitus, *n* (%)	35 (26.7)	39 (23.4)	0.505	37 (27.8)	37 (22.4)	0.284
Hyperlipidemia, *n* (%)	32 (24.4)	37 (22.2)	0.644	32 (24.1)	37 (22.4)	0.739
CAD, *n* (%)	14 (10.7)	15 (9.0)	0.622	12 (9.0)	17 (10.3)	0.711
NIHSS score, median (IQR)	5 (2–8)	3 (1–4)	0.000	4 (2–8)	3 (1–5)	0.001
BI, median (IQR)	65 (40–95)	95 (70–100)	0.000	75 (40–95)	95 (55–100)	0.000
Modified Rankin scale, median (IQR)	3.01 (2–4)	2.22 (1–3)	0.000	2.9 (2–4)	2.29 (1–4)	0.000
SSRS, median (IQR)	39.06 (34–45)	40.3 (34–45)	0.426	39.65 (35–44)	39.84 (34–46)	0.890
Objective support, median (IQR)	9.21 (8–11)	9.37 (8–11)	0.865	9.13 (7.5–11)	9.44 (8–11)	0.504
Subjective support, median (IQR)	23.02 (19–27)	23.81 (20–28)	0.338	23.79 (20–28)	23.2 (19–27)	0.218
Utilization of support, median (IQR)	6.81 (6–8)	7.10 (6–8)	0.294	6.70 (5–8)	7.19 (6–8.5)	0.060
Stroke history, *n* (%)	29 (22.1)	27 (16.2)	0.190	19 (14.3)	37 (22.4)	0.074
TOAST subtypes			0.051			0.596
LAA, *n* (%)	90 (61.4)	93 (55.7)		84 (63.2)	99 (60.0)	
CE, *n* (%)	6 (4.6)	16 (9.6)		11 (8.3)	11 (6.7)	
SAO, *n* (%)	10 (7.6)	22 (13.2)		13 (9.8)	19 (11.5)	
OC, *n* (%)	13 (9.9)	14 (8.4)		8 (6.0)	19 (11.5)	
SUD, *n* (%)	12 (9.2)	22 (13.2)		17 (12.8)	17 (10.3)	
Cerebral atrophy, median (IQR)	0.3 (0.28–0.31)	0.3 (0.28–0.32)	0.905	0.29 (0.27–0.31)	0.3 (0.28–0.32)	0.051
LICA stenosis, *n* (%)	20 (16.0)	31 (18.6)	0.568	22 (16.5)	30 (18.2)	0.711
LMCA stenosis, *n* (%)	35 (26.7)	24 (14.4)	0.008	35 (26.3)	24 (14.5)	0.011
RICA stenosis, *n* (%)	32 (24.4)	30 (18.0)	0.172	33 (24.8)	29 (17.6)	0.126
RMCA stenosis, *n* (%)	36 (27.5)	34 (20.4)	0.150	38 (28.6)	32 (19.4)	0.063
BA stenosis, *n* (%)	11 (8.4)	4 (2.4)	0.019	7 (5.3)	8 (4.8)	0.871
Treatment of stenosis, *n* (%)	12 (9.2)	10 (6.0)	0.299	14 (10.5)	8 (4.8)	0.062

*CAD, coronary artery disease; NIHSS, National Institutes of Health Stroke Scale; BI, Barthel index; SSRS, Social Support Rating Scale; TOAST, Trial of Org 10,172 in acute stroke treatment; LAA, large artery atherosclerosis; CE, cardio-embolic; SAO, small artery occlusion; OC, Other causes; SUD, Stroke of undetermined cause; LICA, left internal carotid artery; LMCA, left middle cerebral artery; RICA, right internal carotid artery; RMCA, right middle cerebral artery; BA, basilar artery*.

**Table 2:** Relationship between lesion location and PSD.

**Variables**	**At discharge**			**At 3 months**		
	**PSD (131)**	**Non-PSD (167)**	***P*-value**	**PSD (133)**	**Non-PSD (165)**	***P*-value**
Left frontal lobe, *n* (%)	12 (9.2)	13 (7.8)	0.671	13 (9.8)	12 (7.3)	0.439
Left temporal lobe, *n* (%)	7 (5.3)	9 (5.4)	0.986	6 (4.5)	10 (6.1)	0.555
Left occipital lobe, *n* (%)	8 (6.1)	11 (6.6)	0.866	9 (6.8)	10 (6.1)	0.804
Left parietal lobe, *n* (%)	11 (8.4)	17 (10.2)	0.601	17 (12.8)	11 (6.7)	0.072
Left insular lobe, *n* (%)	6 (4.6)	5 (3.0)	0.471	5 (3.8)	6 (3.6)	0.955
Left basal ganglia, *n* (%)	25 (19.1)	24 (14.4)	0.276	22 (16.5)	27 (16.4)	0.967
Left thalamus, *n* (%)	8 (6.1)	15 (9.0)	0.356	10 (7.5)	13 (7.9)	0.908
Left corona radiata, *n* (%)	28 (21.4)	29 (17.4)	0.382	29 (21.8)	28 (17.0)	0.291
Left cerebellum, *n* (%)	3 (2.3)	5 (3.0)	0.709	3 (2.3)	5 (3.0)	0.681
Right frontal lobe, *n* (%)	20 (15.3)	22 (13.2)	0.606	17 (12.8)	25 (15.2)	0.559
Right temporal lobe, *n* (%)	18 (13.7)	13 (7.8)	0.095	18 (13.5)	13 (7.9)	0.112
Right occipital lobe, *n* (%)	16 (12.2)	12 (7.2)	0.140	14 (10.5)	14 (8.5)	0.548
Right parietal lobe, *n* (%)	20 (15.3)	16 (9.6)	0.135	18 (13.5)	18 (10.9)	0.489
Right insular lobe, *n* (%)	7 (5.3)	9 (5.4)	0.986	10 (7.5)	6 (3.6)	0.139
Right basal ganglia, *n* (%)	27 (20.6)	44 (26.3)	0.299	34 (25.6)	37 (22.4)	0.527
Right thalamus, *n* (%)	7 (5.3)	15 (9.0)	0.233	8 (6.0)	14 (8.5)	0.418
Right corona radiata, *n* (%)	30 (22.9)	39 (23.4)	0.927	33 (24.8)	36 (21.8)	0.542
Right cerebellum, *n* (%)	4 (3.1)	6 (3.6)	0.797	3 (2.3)	7 (4.2)	0.344
Brainstem, *n* (%)	36 (27.5)	42 (25.1)	0.650	34 (25.6)	44 (26.7)	0.83

Multivariable logistic regression analysis was performed to find the BI [odds ratio (OR) = 0.974, 95% confidence interval (CI) 0.965–0.983, *p* = 0.000], left middle cerebral artery stenosis (OR = 2.118, 95% CI: 1.130–3.968, *p* = 0.019), drinking history (OR = 0.489, 95% CI: 0.241–0.994, *p* = 0.048), basilar artery stenosis (OR = 3.773, 95% CI: 1.080–13.178, *p* = 0.037) was independent and significantly correlated with PSD at discharge (Table 3). BI (OR = 0.989, 95% CI: 0.980–0.997, *p* = 0.011), left middle cerebral artery stenosis (OR = 2.161, 95% CI: 1.181–3.956, *p* = 0.012), female (OR = 2.779, 95% CI: 1.561–4.948, *p* = 0.001) were independently and significantly related with PSD at 3 months after the onset of ischemic stroke (Table 4).

**Table 3:** Predictors of PSD at discharge of stroke.

**Variables**	**β**	***p***	**OR**	**95% CI**
BI LMCA stenosis Drinking history BA stenosis	−0.026 0.750 −0.714 1.328	0.000 0.019 0.048 0.037	0.974 2.118 0.489 3.773	0.965–0.983 1.130–3.968 0.241–0.994 1.080–13.178

*BI, Barthel index; LMCA, left middle cerebral artery; BA, basilar artery; OR, odd ratio; CI, confidence interval*.

**Table 4:** Predictors of PSD at 3 months after stroke.

**Variables**	**β**	***p***	**OR**	**95% CI**
BI LMCA stenosis Gender	−0.011 0.771 1.022	0.011 0.012 0.001	0.989 2.161 2.779	0.980–0.997 1.181–3.956 1.561–4.948

*BI, Barthel index; LMCA, left middle cerebral artery; OR, odd ratio; CI, confidence interval*.

**Figure 2:**
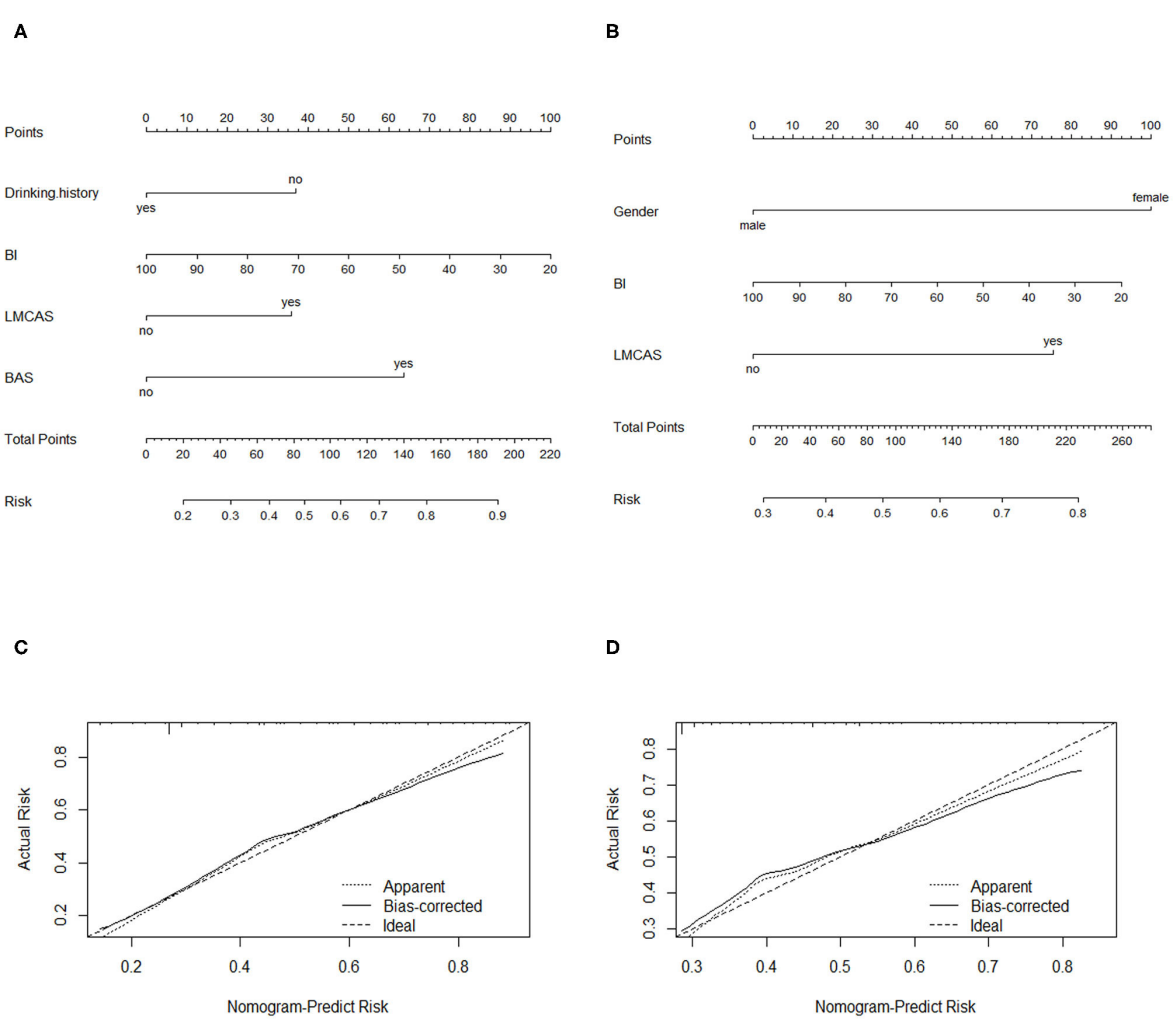
Nomograms of predicting PSD **(A)** at discharge and **(C)** at 3 months after ischemic stroke onset. BI, Barthel index; LMCAS, left middle cerebral artery stenosis; BAS: basilar artery stenosis Calibration plots of the nomograms for PSD prediction of the **(B)** at discharge and **(D)** at 3 months after ischemic stroke onset. X-axis represents the nomogram-predicted probability of depression; Y-axis represents the actual depression probability. A perfectly accurate nomogram prediction model would result in a plot in which the observed and predicted probability for given groups fall along 45-degree line.

Based on the results of the multiple logistic regression analysis, all independent prognostic factors for PSD was brought into the construction of the nomograms as in Figure 2. Each variable was projected up to the value of the small ruler to obtain the score for each parameter. The points were added across independent variables to derive total point, which were converted to predicted probabilities. The nomogram C-index at discharge was 0.736 (95% CI: 0.709–0.763), and the nomogram C-index was 0.689 (95% CI: 0.661–0.717) for 3 months after ischemic stroke onset. In addition, the optimal calibration curve demonstrating the agreements between prediction and actual observation on the presence of PSD at discharge and 3 months after ischemic stroke onset.

## Discussion

This prospective cohort study showed that left middle cerebral artery and basilar artery stenosis were risk factors of PSD. As shown in Table 1, the ratio of left middle cerebral artery and basilar artery stenosis at discharge was much higher than that in non-PSD (26.7 vs. 14.4%, 8.4 vs. 2.4%), and the ratio of left middle cerebral artery stenosis 3 months after ischemic stroke onset in PSD was higher than non-PSD (26.3 vs. 14.5%). This study suggests that cerebral artery stenosis should be considered a potential etiology of PSD.

Previous studies have found that high-grade internal carotid artery stenosis was associated with depressive symptoms (25). This study showed that left middle cerebral artery stenosis was associated with PSD at discharge and 3 months after ischemic stroke onset. It is well-known that the middle cerebral artery supplies blood flow to the frontal, temporal, parietal, caudate nucleus, and the frontal cortex is responsible for emotional regulation. When middle cerebral artery stenosis reaches 50%, hemodynamic disorder and hypoperfusion of specific brain region begin to occur (26). Moreover, clinical studies found that decreased blood flow in the frontal, temporal and parietal lobes was associated with depressive symptoms in the elderly (13, 27, 28). When the patient has chronic cerebral artery stenosis but no ischemic stroke, the hypoperfusion caused by artery stenosis may be compensated by collateral circulation, while decompensation occurs when acute ischemic stroke occurs due to rapid artery occlusion. Due to the brain's higher energy requirements and lower energy reserves, when hypoperfusion occurs, the supply of glucose and oxygen became insufficient, resulting in metabolic toxicity (29, 30). Neuronal dysfunction of sympathetic nerve fibers may lead to an imbalance of serotonergic or noradrenergic neurotransmitter associated with depression.

The findings of the study indicate that the left middle cerebral artery stenosis increased the risk of PSD might associate with the dominant hemisphere of the left hemisphere. The left frontal lobe is associated with positive emotions and well-being, while the right frontal lobe is associated with negative emotion regulation (31–33). As a result, hypoperfusion in the left frontal lobe caused by left middle cerebral artery stenosis was more likely to cause depressive symptoms.

In this study, internal carotid artery stenosis was not related to PSD. However, previous studies have shown that internal carotid artery stenosis was associated with depression, with artery stenosis above 80% as the cut-off value (12). Previous studies had shown that when the internal carotid artery stenosis was >70%, the blood flow decreased significantly with the increase of stenosis degree (34). According to 2003 Radiological Society of North America (RSNA) Annual Meeting's carotid artery stenosis diagnostic criteria issued by the ultrasound division, hemodynamic disorders occur when the internal carotid artery stenosis was above 50%, and obvious hemodynamic disorders appear when internal carotid artery stenosis was above 70%. Given that the internal carotid artery was larger in diameter, no obvious hemodynamic disorder and hypoperfusion occurred when 50% was used as the cut-off value, which might be the reason why there was no association found between internal carotid artery stenosis and depression.

Interestingly, in this study, the basilar artery was associated with PSD at discharge. When basilar artery stenosis occurs, chronic ischemia occurs in the cerebellum and brainstem. Previous literatures found that the cerebellum plays an important role in emotion regulation (35–37). Jiang et al. (38) reported that patients with depression experience abnormal connections in the prefrontal-thalamic-cerebellar circuits. The “remote effects” of cerebellar or brainstem damage may be related to the occurrence of PSD, with supratentorial damage in one cerebral hemisphere leading to decreased metabolism in the contralateral cerebellar hemisphere. Therefore, damage to one side of the cerebellar hemisphere can also lead to a decline in the metabolism of the frontal cortex, and the dysfunction of the frontal cortex may also lead to depression (39). The disappearance of depression 3 months after stroke may be related to the shorter duration of low metabolism (40), and the normal metabolism of the frontal lobe can be restored by compensating of the surrounding brain tissue (41).

Previous studies have found that the location of lesion was related to PSD (42–44), such as prefrontal, temporal lobe, etc. However, in this study the association between the lesion location and the onset of depression was not found, may be caused by including patients with lesion volume varied from lacunar infarction to large cerebral infarction, and there was a correlation between lesion volume and severity of depression (45, 46). This study showed that patients with higher BI scores had lower rates of depression, which indicated that better recovery of limb function had lower rates of depression. Previous studies have found that the degree of limb function recovery was related to the occurrence of PSD (47–49). The BI scores also measures the ability of daily living after stroke. Higher BI score indicated better recovery of limb function (50).

In addition, PSD patients at discharge in this study had lower rates of drinking history than non-PSD patients (11.5 vs. 20.5%), indicating that moderate alcohol consumption was a protective factor for PSD (51–53). The mental effects of alcohol were mainly due to GABA (gamma-aminobutyric acid) receptor-mediated sedation, disinhibition, relaxation and negative thoughts suppression, the opiate-mediated reward neurotransmitter systems that lead to positive feedback of alcohol consumption (54, 55). However, the effects of alcohol disappeared 3 months after stroke, probably due to a stoppage of drinking for most patients after stroke. In this study, female had a higher risk of depression than male at 3 months after stroke. Epidemiological studies showed that the risk of depression was 2.5 times higher in female than male (56). To make matters worse, since the average age of female in this study was 57.90 ± 9.89, female patients also faced an increased risk of postmenopausal depression. According to the study of menopausal depression symptoms among Chinese women, postmenopausal (56.6 ± 4.9) depression rates were higher than premenopausal (45.3 ± 3.2) and menopausal (48.6 ± 3.4) depression (57).

This was a prospective cohort study and the first to show that stenosis of the left middle cerebral artery and basilar artery were associated with PSD. In the future treatment of cerebrovascular diseases and mood disorders, the potential impact of artery stenosis on mood should be considered. While focusing on stroke recurrence in future studies, the sample size of endovascular therapy should be further increased to observe the potential effect of endovascular therapy on mood. Normogram is widely used in surgical risk assessment, tumor prognosis and survival analysis (58–61). As far as the current prediction models are concerned, the nomogram that are easy for clinicians to use has good accuracy and discrimination power for the prediction results, which is helpful for clinical decision-making. We established two normograms with good predictive effect, which could accurately predict the PSD risk at discharge and 3 months, contributing to the early identification of high-risk PSD population and timely adoption of preventive or therapeutic measures to promote the recovery of patients after stroke.

As for advantages of this study. Firstly, being the first to show association between the stenosis of the left middle cerebral artery and basilar artery with PSD, this prospective cohort study was able to investigate the causal relationship between arterial stenosis and PSD. Also, this study included social demographics, risk factors of cerebrovascular disease and locations of ischemic strokes to rule out the influence of confounding factors.

As for some limitations that should be considered. Firstly, there may be errors in the manual measurement of artery stenosis ratio, and software measurement based on computer technology can improve the accuracy in the future. Secondly, the sample size was small, patients who did not complete CTA or MRA, aphasia, or deafness were not included in the study, which might increase selection bias. Thirdly, we did not include the lesions size and apraxia into the analysis, which are important factors affecting PSD (62). In our future research, we will combine artificial intelligence, brain atlas and voxel-based analysis to analyze the influence of the lesion location and lesions size on PSD. Lastly, other cerebral arteries were not included in the evaluation due to measurement difficulties.

## Conclusion

An association was found between left middle cerebral artery stenosis and basilar artery stenosis, BI score, gender and drinking history and PSD. For the patients with stroke, early rehabilitation was particularly important. PSD significantly affects the rehabilitation and quality of life of the patients. Therefore, early identification of PSD and appropriate preventive measures are particularly important. This finding may be conducive to the detection of high-risk patients and early intervention to promote full recovery. Importantly, this study suggested that the potential impact of artery stenosis on mood should be considered, which may be a potential new approach for PSD treatment. In the future, studies with the larger sample size of endovascular treatment should be conducted to observe the potential effect of endovascular treatment on PSD

## Data Availability Statement

The datasets presented in this article are not readily available because further data mining is ongoing. Requests to access the datasets should be directed to 15738862357@163.com.

## Ethics Statement

The studies involving human participants were reviewed and approved by the ethics committee of Tongji Medical College, Huazhong University of Science and Technology. The patients/participants provided their written informed consent to participate in this study.

## Author Contributions

SZ and ZZ led the study. XQ performed the data analysis and implemented the methodology. XS, WS, JM, YL, GL, and XZ collected the data. ZZ and XQ prepared the original draft. SZ reviewed and edited the final manuscript. YC touched the language of this article. All authors contributed to the article and approved the submitted version.

## Conflict of Interest

The authors declare that the research was conducted in the absence of any commercial or financial relationships that could be construed as a potential conflict of interest.
