# Supplementary material for: Association of Cerebral Artery Stenosis With Post-stroke Depression at Discharge and 3 Months After Ischemic Stroke Onset
Source: Front Psychiatry. 2020 Nov 25;11:585201. doi: 10.3389/fpsyt.2020.585201 (PMC7723904; doi:10.3389/fpsyt.2020.585201)
Supplement: Supplementary file 1 [file Table_1.DOCX]

To test whether vascular recanalization therapy can reduce the incidence of PSD. A total of 22 patients received vascular recanalization (thrombolytic therapy / stent implantation). Patients with artery stenosis were grouped for vascular recanalization and Mann–Whitney U test was performed for depression score (Table 3).No improvement in PSD by vascular recanalization was found.

Table Patients with arterial stenosis depression score at discharge and 3 months after ischemic stroke onset

| Variables | Number of cases | | PSD at discharge | | | PSD at 3 months | | |
| --- | --- | --- | --- | --- | --- | --- | --- | --- |
|  | vascular recanalization | non vascular recanalization | vascular recanalization | non vascular recanalization | P value | vascular recanalization | non vascular recanalization | P value |
| LICA stenosis  LMCA stenosis  RICA stenosis  RMCA stenosis  BA stenosis | 4  6  4  6  2 | 47  53  58  64  13 | 10.50（7.78）  11.75（4.57）  14.33（8.82）  11.20（3.56）  12.50（4.95） | 7.74（5.36）  8.71（4.80）  8.69（6.27）  8.46（5.40）  10.54（5.62） | 0.416  0.217  0.177  0.193  0.669 | 10.50（2.12）  9.50（5.97）  10.00（1.73）  9.00（3.32）  7.00（7.07） | 7.50（4.59）  9.30（4.48）  8.85（5.67）  8.57（5.91）  11.15（7.44） | 0.241  0.905  0.420  0.615  0.491 |
